# Supplementary figures and images for: B-1 cell-mediated modulation of M1 macrophage profile ameliorates microbicidal functions and disrupt the evasion mechanisms of Encephalitozoon cuniculi
Source: PLoS Negl Trop Dis. 2019 Sep 19;13(9):e0007674. doi: 10.1371/journal.pntd.0007674 (PMC6779274; doi:10.1371/journal.pntd.0007674)

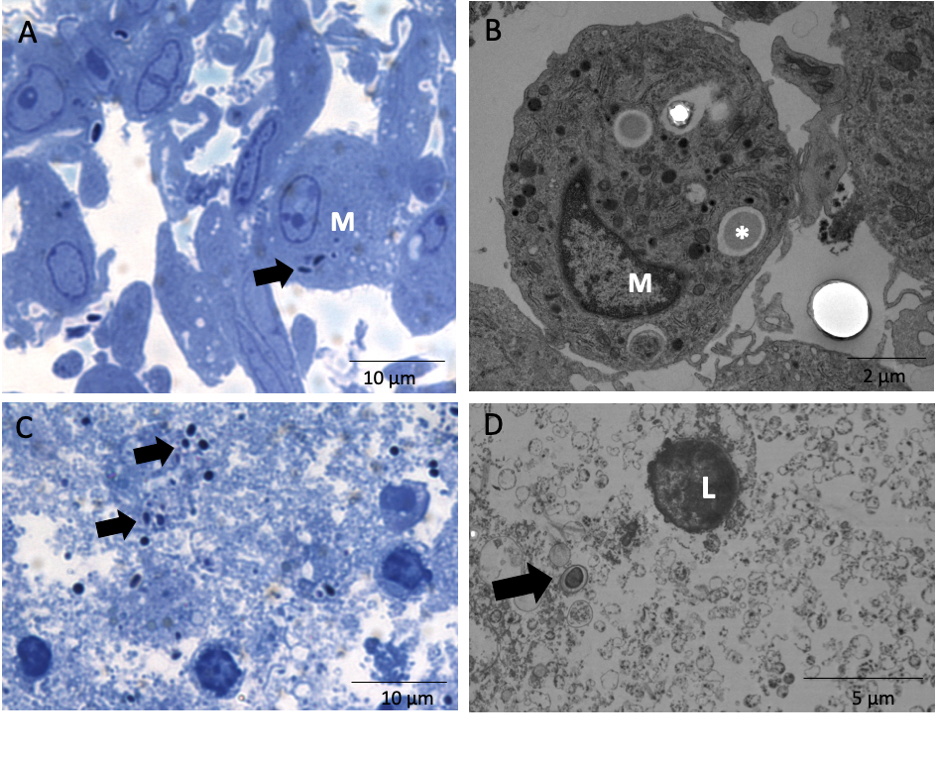

Supplement: S1 Fig — (A) Photomicrography of phagocytic cells with E. cuniculi spores inside them (arrow) in APerC XID with144 h of infection. (B) Ultramicrography of a phagocytic cell with E. cuniculi spores in lysis (arrow) in APerC XID after 144 h of infection. (C) Photomicrography of APerC BALB/c in the absence of macrophages and mature spores of E. cuniculi outside the cells (arrow) and degenerated lymphocytes (head arrow) after 144 h of infection. (D) Ultramicrography of APerC BALB/c with the absence of macrophages and mature spores of E. cuniculi outside the cells (arrow) and degenerated lymphocytes (head arrow) with pyknotic nucleus after 144 h of infection. (TIFF) [file pntd.0007674.s001.tiff]
